# Influence of maternal and socioeconomic factors on breast milk fatty acid composition in urban, low‐income families

**DOI:** 10.1111/mcn.12423

**Published:** 2017-02-15

**Authors:** Uma Nayak, Suman Kanungo, Dadong Zhang, E. Ross Colgate, Marya P. Carmolli, Ayan Dey, Masud Alam, Byomkesh Manna, Ranjan Kumar Nandy, Deok Ryun Kim, Dilip Kumar Paul, Saugato Choudhury, Sushama Sahoo, William S. Harris, Thomas F. Wierzba, Tahmeed Ahmed, Beth D. Kirkpatrick, Rashidul Haque, William A. Petri, Josyf C. Mychaleckyj

**Affiliations:** ^1^ Center for Public Health Genomics University of Virginia Charlottesville 22908 Virginia USA; ^2^ National Institute of Cholera and Enteric Diseases Kolkata India; ^3^ International Center for Diarrhoeal Disease Research Dhaka Bangladesh; ^4^ Department of Medicine and Vaccine Testing Center University of Vermont College of Medicine Burlington Vermont USA; ^5^ International Vaccine Institute Seoul South Korea; ^6^ Dr. B.C. Roy Post Graduate Institute of Paediatric Sciences Kolkata India; ^7^ OmegaQuant Analytics Sioux Falls South Dakota USA; ^8^ Division of Infectious Diseases and International Health University of Virginia Charlottesville 22908 Virginia USA; ^9^ Department of Pathology University of Virginia Charlottesville Virginia USA 22908; ^10^ Department of Public Health Sciences University of Virginia Charlottesville 22908 Virginia USA

**Keywords:** anthropometry, breast milk, infant growth, low‐income countries, polyunsaturated fatty acids, socioeconomic factors

## Abstract

The lipid composition of breast milk may have a significant impact on early infant growth and cognitive development. Comprehensive breast milk data is lacking from low‐income populations in the Indian subcontinent impeding assessment of deficiencies and limiting development of maternal nutritional interventions. A single breast milk specimen was collected within 6 weeks postpartum from two low‐income maternal cohorts of exclusively breastfed infants, from Dhaka, Bangladesh (*n* = 683) and Kolkata, India (*n* = 372) and assayed for percentage composition of 26 fatty acids. Mature milk (>15 days) in Dhaka (*n* = 99) compared to Kolkata (*n* = 372) was higher in total saturated fatty acid (SFA; mean 48% vs. 44%) and disproportionately lower in ω3‐polyunsaturated fatty acid (PUFA), hence the ω6‐ and ω3‐PUFA ratio in Dhaka were almost double the value in Kolkata. In both sites, after adjusting for days of lactation, increased maternal education was associated with decreased SFA and PUFA, and increasing birth order or total pregnancies was associated with decreasing ω6‐PUFA or ω3‐PUFA by a factor of 0.95 for each birth and pregnancy. In Dhaka, household prosperity was associated with decreased SFA and PUFA and increased ω6‐ and ω3‐PUFA. Maternal height was associated with increased SFA and PUFA in Kolkata (1% increase per 1 cm), but body mass index showed no independent association with either ratio in either cohort. In summary, the socioeconomic factors of maternal education and household prosperity were associated with breast milk composition, although prosperity may only be important in higher cost of living communities. Associated maternal biological factors were height and infant birth order, but not adiposity. Further study is needed to elucidate the underlying mechanisms of these effects.

**Abbreviations**
AA: Arachidonic acid
ALA: α‐Linolenic acid
ARA: Arachidic acid
BEH: Behenic acid
BMI: Body mass index
CAP: Capric acid
DGLA: Dihomo‐γ‐linolenic acid
DHA: Docosahexaenoic acid
DPA6: Docosapentaenoic‐n6 acid
DTA: Docosatetraenoic acid
EPA: Eicosapentaenoic acid
FA: Fatty acid
GLA: γ‐Linolenic acid
LASSO: Least Absolute Shrinkage and Selection Operator
LAU: Lauric acid
LIG: Lignoceric acid
LLA: Linoelaidic acid
MUFA: Monounsaturated fatty acid
MYR: Myristic acid
NER: Nervonic acid
OLE: Oleic acid
PAL: Palmitic acid
PROVIDE: Performance of Rotavirus and Oral Poliovirus Vaccines in Developing Countries
PUFA: Polyunsaturated fatty acid
SES: Socioeconomic status
SFA: Saturated fatty acid
STE: Stearic acid
TFA: trans‐fatty acid

## 1 INTRODUCTION

Exclusive breast‐feeding is the preferred method of feeding during the first 6 months of age to support optimal growth and development and to protect against gastrointestinal disease, diarrhea, and respiratory tract infection. It is the reference model against which all alternative‐feeding methods are measured with regard to growth, health, development, and all other short‐and long‐term outcomes (Gartner et al., 2005). In recent years, extensive research has been directed towards the lipid component of breast milk, which provides not only calories and macronutrition but also key micronutrients for infant growth and cognitive development. Docosahexaenoic acid (DHA) and arachidonic acid (AA) are vital polyunsaturated fatty acids (PUFAs) in the neuron‐rich grey matter of the brain (Lauritzen & Carlson, 2011). Although there is evidence that fetuses and preterm infants are able to endogenously synthesize AA and DHA, the synthesis is extremely low (Uauy, Mena, & Rojas, 2000) making maternal long‐chain PUFA supply critical during fetal and postnatal growth and development. In Bangladesh, the national median breast‐feeding duration is 31.2 months (National Institute of Population Research and Training [NIPORT], 2013) making it a vital source of neonatal energy, fat, and other nutrients. Research conducted in western countries has greatly expanded knowledge of the biological effects of fatty acid (FA) composition; the role of essential FAs on infant growth, neurodevelopment, visual acuity, and gut integrity; and the epidemiological factors affecting breast milk composition (Fleith & Clandinin, 2005; Qawasmi, Landeros‐Weisenberger, & Bloch, 2013; Teitelbaum & Walker, 2001). The work in lower‐income countries has been more limited, particularly in the Indian subcontinent, and there is a dearth of well‐powered studies investigating the epidemiological factors that affect the composition of breast milk and hence potentially, the development and health of the infant. In populations with food insecurity, breast milk fat content may be suboptimal (Jensen, 1999; Brown, Akhtar, Robertson, & Ahmed, 1986), but the variables and mechanisms affecting breast milk fatty acid (FA) composition are not well understood.

In order to help fill this gap, we designed this analysis to (a) describe and compare the breast milk FA profiles in cohorts from low‐income populations in Bangladesh and India up to 6_weeks postpartum, using data from an ongoing research program to evaluate vaccine performance, environmental enteropathy, and infant development in these countries; and (b) examine the association of maternal and socioeconomic factors collected in the study with the composition measured as ratios of major FA percentages. Additionally, we describe the implementation in the field of a new convenient dried milk spot protocol to determine breast milk composition, which allows easy transportation of large numbers of samples from remote field study areas to a central laboratory for FA determination.

**Key messages**

- Little comprehensive breast milk composition data exists from the Indian subcontinent, hence we assayed 26 FAs in >1,000 mothers in two low‐income, urban cohorts in Dhaka, Bangladesh and Kolkata, India.
- Clinical and demographic data revealed better SES and nourishment for mothers in Kolkata compared to Dhaka.
- Dhaka milk was higher in total SFA and lower in ω3‐PUFA compared to Kolkata, and contained almost double the ω6‐PUFA/ω3‐PUFA ratio of Kolkata.
- Socioeconomic factors associated with composition included increased maternal education (both sites) and increased household prosperity (Dhaka only).
- Associated maternal biological factors were maternal height and increasing infant birth and pregnancy order in both sites, but postpartum maternal BMI was not associated.

## 2 MATERIALS AND METHODS

### 2.1 Study population

The clinical characteristics and design of the cohort for the Dhaka, Bangladesh site have been reported previously (Kirkpatrick et al., 2015). Briefly, mothers and infants were recruited as part of the performance of rotavirus and oral poliovirus vaccines in developing countries (PROVIDE) study, conducted in two sites in Dhaka, Bangladesh and Kolkata, India. The mothers were not subject to vaccine trial intervention in either site and hence the trial structure will be ignored here. They constituted two prospective maternal cohorts, randomly recruited subjects to the family exclusion and inclusion criteria (supplemental Table 1) for the vaccine trials, with no additional eligibility criteria for the breast milk substudy. In Bangladesh, 700 mothers from low‐socioeconomic households in the slum areas of the Mirpur Thana of Dhaka and newborns were enrolled into PROVIDE between May 2011 and November 2012. They were consented within 7 days postdelivery after confirming eligibility to participate, and their intention to comply with study protocol and to remain living in the study enrollment area. At the India study site, 372 mother–infant pairs dwelling in the urban slums were enrolled at the infant week 6 expanded program on immunization visit to Dr. B.C. Roy Post Graduate Institute of Paediatric Sciences in Kolkata, India, from March 2012 to October 2013. The studies were approved by the ethical review committee for human subjects protection and research review committee for scientific merit at the International Centre for Diarrhoeal Diseases research, Dhaka, Bangladesh; Institutional Ethical Committee at National Institute for Cholera and Enteric Diseases, Kolkata, India; and Institutional Review Boards at the International Vaccine Institute, South Korea, University of Virginia and University of Vermont.

### 2.2 Anthropometry

In Bangladesh, maternal anthropometric measurements were performed during the 6th week study visit by trained field researchers. Maternal weight was measured using a Tanita analog‐dial‐scale to the nearest 10 g, and height was measured using a vertical measuring board with an attached tape measure to the nearest 0.1 cm. In India, the measurements were taken at their enrollment visit at 6 weeks postpartum, by trained personnel in presence of the physicians in the study clinic at the hospital.

### 2.3 Socioeconomic status (SES) and demographic information

At enrollment, a detailed questionnaire on family SES and demographic information was administered to the mothers in their homes by a field research assistant in Bangladesh and at the children's hospital clinic in India.

### 2.4 Infant gestational age

The gestational age of the neonates was estimated on a subset of the infants in the Bangladesh site to distinguish fetal growth restriction from prematurity (<37 weeks gestational age) using the Dubowitz‐Ballard assessment scale (Ballard, Novak, & Driver, 1979). This data was not collected in India.

### 2.5 Breast milk sample collection

A single breast milk specimen was collected within a target period of up to 6 weeks postpartum from each mother, but with variances because of missed and rescheduled study visits. In Bangladesh, samples were collected during home visits by a trained field research assistant between birth and 6 weeks postpartum. In India, they were collected at the study enrollment visit to the B.C. Roy hospital clinic in the presence of study nurses, at 6 weeks postpartum. In both sites, the mothers were guided to manually express approximately 5 mL breast milk from the breast of their choice and precleansed nipple into a prelabeled falcon tube in the presence of study staff. Samples were collected without restriction to fore or hind milk, or specific time of the day. The samples collected from the field were transported the same day to the laboratories at the International Centre for Diarrhoeal Diseases research, Dhaka, Bangladesh and the National Institute for Cholera and Enteric Diseases in insulated carriers with cold packs at 4°C. In the laboratories, 1 mL of breast milk was stored at −70°C without antioxidant for a mean duration of 14.8 months in Bangladesh, and 14 months in India prior to spotting and shipment to the OmegaQuant Analytics laboratory.

### 2.6 Preparation of dried breast milk spots

For each sample, 1 μL of thawed milk was spotted and dried on a separate filter paper (Ahlstrom 226, PerkinElmer, Greenville, SC) pretreated with an antioxidant cocktail (Oxystop®, OmegaQuant Analytics, Sioux Falls, SD) to protect PUFAs from oxidation. The milk spot cards were shipped to OmegaQuant Analytics laboratory, Sioux Falls, South Dakota, for analysis. The stability of dried breast milk spots and reproducibility has been tested by OmegaQuant (Jackson, Polreis, Sanborn, Chaima, & Harris, 2016). Focusing on DHA as the most highly unsaturated FA in the sample, the dried milk spots have been shown to be stable for at least 4 weeks at room temperature, and up to 3 years at −80°C. All measured percentage DHA levels were within 15% of the referent value.

### 2.7 Breast milk FA analysis

At OmegaQuant, a punch from the dried milk spot was placed in a vial containing 0.5 mL of methylating reagent (boron trifluoride in methanol [14%] and toluene and methanol [35/30/35 v/v]). The vial was briefly shaken and heated at 100°C for 45 min. After cooling, hexane and distilled water (0.5 mL each) were added, and samples were spun to separate layers. An aliquot of the hexane (upper) layer containing the FA methyl esters were measured by gas chromatography as described previously (Harris, Pottala, Vasan, Larson, & Robins, 2012). Individual breast milk FAs were expressed as percentage wt/wt of total identified FA; the FA profile of each specimen contained 26 individual FAs.

### 2.8 Statistical analyses

Descriptive statistics of continuous variables and individual breast milk FA percentages were expressed as means ± standard deviations. To compare the FA profiles of variable lactation duration Bangladesh samples with the solely mature breast milk samples collected in India, we applied stratification to days of lactation in Bangladesh to include only mature milk samples (>15 days pospartum, *N* = 99). Equality of means of FA fractions between sites was tested by Welch's *t* test. The large sample sizes in this study mean that the skewed FA distributions can be ignored for the purpose of testing correlations and equality of means under the asymptotic central limit theorem. The missing FA data for 17 of 700 Bangladesh mothers were tested for informative dropout by univariate logistic regression of the missing indicator variable on each of the seven model explanatory variables (Results and Tables 3 and 4). Summary level analyses of the 26 FA compositions used major FA fractions created by summation of the constituent single FA molecular moiety percentages into (a) saturated fatty acid (SFA); (b) PUFA; (c) ω6‐PUFA; (d) ω3‐PUFA; (e) monounsaturated fatty acid (MUFA); and (f) trans‐FAs. The overall complexity of the FA mixture in each breast milk sample was summarized as a single variable using Shannon entropy (*H*; Shannon, 1948), computed as

$$H=-\sum_{i=1}^{26}p\left(x_{i}\right)\log\left(p\left(x_{i}\right)\right),$$

where *p*(*x*_*i*_) is the proportion of the *i*th single constituent FA in the FA profile. The Shannon Index (*H*) can vary from zero complexity (only a single FA present at 100%) to a maximum complexity of log (26) = 3.26 when all FAs are present in equal proportions. A higher *H* value indicates a more uniform distribution of FAs in a sample (higher mixture “complexity”).

We computed log ratios of major fractions for two primary outcomes that we wished to test for association with maternal and socioeconomic factors: log (SFA/PUFA) and log (ω6‐PUFA/ω3‐PUFA). The value of ratios versus individual percentage fractions is that they allow measurement of the change in partitioning of FAs between the two fractions. When individual fractions are tested, the 100% compositional constraint implies the sum of other fractions must oppositely change in total, but does specify how the change is distributed. A priori, economic security and wealth were expected to be strong candidate explanatory factors in these populations, but adjusting for currency differences and cost of living between countries is complex and unlikely to be fully captured by simple national gross domestic product scaling. Instead, a prosperity index was developed using data from a common set of interview questions across both Bangladesh and India sites that collected information on tangible or durable household assets, and occupation. Twenty‐nine common study variables were identified, scaled, and used in a principal component analysis using joint data from both sites (supplemental Table 2). The resulting principal components were candidate prosperity indices that could be used as explanatory factors for both sites. Principal component 1 (prosperity index 1) had a significantly higher correlation with household expenditure than the other 28 in Bangladesh and India (supplemental Table 3).

Association of maternal and socioeconomic factors with breast milk composition was tested using a two‐step selection strategy to control possible overfitting from many potential explanatory factors. The two breast milk composition outcomes were the two log ratios described above. The first step identified a limited set of five prespecified candidate explanatory factors identified from previously published articles (Antonakou et al., 2013; Jensen, 1999; Prentice, Jarjou, Drury, Dewit, & Crawford, 1989): maternal age, height, postnatal body mass index (BMI), education, and age of the infant at breast milk collection (because breast milk samples in Bangladesh were collected between 2–43 days postdelivery). Maternal height and anthropometry were important to test whether breast milk composition could be at least partially responsible for the transmission of linear growth deficits from mother to infant in a cycle of malnutrition.

These five prespecified variables were supplemented in the second step using penalized least absolute shrinkage and selection operator (LASSO) variable selection (Lockhart, Taylor, Tibshirani, & Tibshirani, 2014; Tibshirani, 1996) from 28 candidate variables in Bangladesh and 27 in India (supplemental Table 4). The LASSO algorithm selects the most significant associated individual variables with the outcome in steps, simultaneously adjusting for testing multiple variables. LASSO‐selected predictors for inclusion in multiple regression models were required to meet a significant level of 0.05 under the covariance test that adjusts the degrees of freedom for competing multiple predictor selection (Lockhart et al., 2014). Any LASSO‐selected predictor in any of the two analyses (outcomes) was included in all multiple linear regression models for that site. More technical details are available in supplemental Methods. We then tested the five pre‐specified and any LASSO‐selected candidate predictors jointly in a multiple linear regression model for each of the two log ratio outcomes in Bangladesh and India separately. We used all maternal samples in Bangladesh (*n* = 683) in these models despite the variable lactation stage, although we found no statistically significant changes in the effect of any of the explanatory variables with days of lactation in Bangladesh (all interaction tests *p* values greater than .05, described in supplementary Methods). The two‐sided statistical significance level was set at α = 0.05 but was adjusted for multiple testing. For statistical comparisons of the 26 individual FA compositions between sites, a statistical test with *p* value less than .05 was considered “suggestive” of association, and a test with *p* value less than .004 (0.05/11.6 effective independent tests) was considered significant taking into account the pairwise correlations of the 26 FAs (supplemental Methods). For the two multiple linear regression models per site, a *p* value less than .05 for a predictor in one model was considered suggestive of association, and a *p* value less than .01 was considered significant (.05 corrected for approximately 5 independent predictors per model). A predictor with a *p* value of less than .05 in both site cohorts was considered significant by replication.

## 3 RESULTS

Of the mothers enrolled in the Bangladesh (700) and India (372) sites of the PROVIDE study, we assayed breast milk FA profiles (26 FAs) for 683/700 (97.6%) and 372/372 (100%), respectively. In Bangladesh, 7 mothers withdrew early from the study and quantity was not sufficient in 10 samples (supplemental Figure 1). The small number of missing FA outcome profiles (*N* = 17, 2.4%) were not found to be informatively missing when regressed on the 7 main predictor variables in Tables 3 and 4 (all *p* values greater than .05) and therefore were ignored. The clinical characteristics of the study participants from Bangladesh and India are shown in Table 1. Comparing Bangladesh to India, the mothers were older (mean age 24.6 vs. 23.5), taller, and had lower postpartum BMI (mean 21.8 vs. 22.9), with 19% versus 9% considered underweight and 63% versus 66% falling in the normal BMI range (18.5–24.9). Mothers in Bangladesh were also less well educated with 66% having either none or less than 5 years of education. The clinical and demographic data suggested better socioeconomic status and better nourishment for mothers in India compared to Bangladesh. Almost one third of the neonates in Bangladesh were estimated to be preterm (32.6%), but the degree of prematurity was mild with a mean gestational age of 37.6 and 91.9% of the preterm infants estimated as 36 weeks. This data was not available in India.

**Figure 1:**
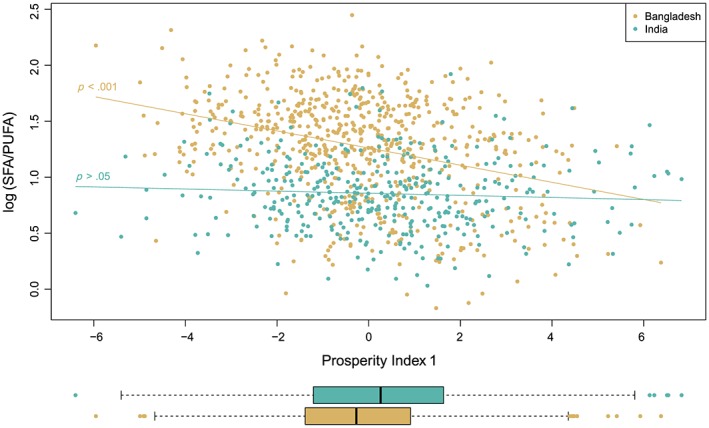
Relationship of family prosperity index 1 with log (SFA/PUFA) for PROVIDE (Performance of Rotavirus and Oral Poliovirus Vaccines in Developing Countries) Study Bangladesh and India site cohorts. Brown icons are for Bangladesh (*n* = 683), blue icons for India (*n* = 372). The color‐coded straight lines are the univariate global linear regression fits to the separate cohort data with annotated *p*‐value that indicates the significance of the Wald test of non‐zero gradient of the fit. The horizontal boxplots under the x‐axis show the distributions of the prosperity index for the two sites, brown fill for Bangladesh, and blue fill for India. The limits of the colored boxes define the interquartile range (1st to 3rd data quartiles), the black solid vertical line in each box is the position of the median prosperity index, and the dotted ranges (whiskers) show the limits of median +/− 1.5 times the interquartile range. The points that lie outside the dotted ranges are putative outliers. SFA = saturated fatty acid; PUFA = polyunsaturated fatty acid

**Table 1:** Clinical characteristics of the mothers with breast milk fatty acid profiles

	Bangladesh	India	*p* value
*n*	683	372	
Age, year	24.6 ± 4.6	23.5 ± 4.2	<0.001
Breast milk sample			
Days since delivery, mean	10.5 ± 6.3	44.8 ± 2.0	<0.001
Days since delivery, range	2–43	42–49	
Anthropometry			
Height, cm	150.3 ± 5.5	146.6 ± 5.3	<0.001
Postpartum weight, kg	49.4 ± 9.4	49.4 ± 9.7	0.907
Postpartum BMI, kg/m^2^	21.8 ± 3.7	22.9 ± 3.9	<0.001
Underweight (<18.5)	122 (18.5)	35 (9.4)	<0.001
Normal (18.5–24.9)	413 (62.7)	245 (65.9)	0.306
Overweight (25.0–29.9)	101 (15.3)	63 (16.9)	0.497
Obese (≥30)	23 (3.5)	29 (7.8)	0.002
Socioeconomic			
Stay home mother, %	589 (86.2)	355 (95.4)	<0.001
First‐live delivery, %	263 (38.5)	193 (46.0)	<0.001
Education, %			
None	196 (28.7)	53 (14.2)	<0.001
Primary	254 (37.2)	97 (26.1)	<0.001
Secondary	124 (18.2)	96 (25.8)	<0.001
Higher	109 (16.0)	126 (33.9)	<0.001
Estimated infant gestational age			
*n*	374	N/A	
Age < 37 weeks, %	122 (32.6)	N/A	
Age, weeks	37.6 ± 1.4	N/A	
Monthly household			
Income, 1,000 currency units^a^	12.8 ± 9.5	N/A	
Expenditure, 1,000 currency units^a^	11.6 ± 7.3	6.0 ± 3.2	<0.001

Values represent mean ± SD or *n* (%).

a During the study period 1 USD = approximately 80 Bangladesh Taka or 50 Indian Rupees.

Equality of mean between sites was tested by Welch's *t* test.

Considering the age of infant at breast milk sampling was 2–43 days in Bangladesh and 42–49 days in India, we compared the FA composition during three phases of lactation in Bangladesh to test for changes between colostrum (1–5 days postpartum), transitional (6–15 days postpartum), and mature milk (more than 15 days postpartum; supplemental Table 5). As lactation proceeded, we found significant changes in means of 19/26 FAs fractions from colostrum to mature milk (*p* value greater than .004) including percentage AA and DHA, while the means of the major summary fraction percentages (SFA, PUFA, ω6‐PUFA, and ω3‐PUFA) and the mean Shannon index of overall mixture composition did not change (*p* value greater than .004). Notably, the ω6‐PUFA/ω3‐PUFA did significantly increase. The correlation between the major summary fractions is shown in supplemental Figure 2 for Bangladesh and India. In both sites, SFA was negatively correlated with PUFA and ω6‐PUFA (−0.70). SFA and MUFA were also negatively correlated (−0.54 in Bangladesh and −0.70 in India). There was a greater difference in the correlation between ω6‐PUFA and ω3‐PUFA (0.76 in Bangladesh and 0.19 in India).

Table 2 compares the FA profiles in mature breast milk from Bangladeshi and Indian mothers. We found significant differences in 21/26 FAs. In Bangladesh, the major FA classes accounted for about 48% (SFA), 37% (MUFA), and 15% (PUFA) compared to 44%, 37%, and 19%, in India (*p* value less than .001 for SFA and PUFA major fractions). Mean percentage GLA was the only PUFA of seven ω6‐PUFAs and four ω3‐PUFAs that was not different, and all four ω3‐PUFAs and percentage linoleic acid, eicosadienoic, and AA where significantly higher in Indian breast milk samples. Although both mean total ω6‐PUFA and ω3‐PUFA were higher in India, the more than doubled ω3‐PUFA resulted in a mean ratio ω6‐PUFA/ω3‐PUFA of 13.4 in Bangladesh, which was 1.8 times higher than India (7.4). The Shannon index in mature Bangladesh breast milk samples was statistically significantly lower (1.9 vs. 2.0) than in India, suggesting that the breast milk samples in India contain a more uniform distribution of FA proportions compared to Bangladesh.

**Table 2:** Percentage composition of breast milk fatty acid in mature milk (>15 days postpartum) between sites^a^

Fatty Acids^b^		Bangladesh	India	*p*‐value
*n*		**99**	**372**	
Capric (CAP)	C10:0	1.3 ± 0.4	1.0 ± 0.4	<0.001
Lauric (LAU)	C12:0	8.5 ± 2.9	6.6 ± 2.3	<0.001
Myristic (MYR)	C14:0	7.8 ± 3.3	7.5 ± 2.9	0.44
Palmitic (PAL)	C16:0	26.4 ± 4.0	24.0 ± 3.1	<0.001
Stearic (STE)	C18:0	3.9 ± 0.8	4.3 ± 0.9	<0.001
Arachidic (ARA)	C20:0	0.1 ± 0.03	0.2 ± 0.06	<0.001
Behenic (BEH)	C22:0	0.07 ± 0.03	0.1 ± 0.04	<0.001
Lignoceric (LIG)	C24:0	0.06 ± 0.02	0.06 ± 0.02	0.85
∑ SFA		48.0 ± 6.8	43.7 ± 5.7	<0.001
Palmitoleic (PLE)	C16:1ω7	3.0 ± 1.0	2.5 ± 0.9	<0.001
Oleic (OLE)	C18:1ω9	33.4 ± 4.9	32.4 ± 3.7	0.025
Eicosenoic (EIC9)	C20:1ω9	0.4 ± 0.2	1.2 ± 0.8	<0.001
Nervonic (NER)	C24:1ω9	0.09 ± 0.07	0.6 ± 0.3	<0.001
**∑** Cis‐MUFA		36.8 ± 5.3	36.6 ± 3.9	0.65
Linoleic (LA)	C18:2ω6	11.7 ± 5.7	14.6 ± 3.4	<0.001
Eicosadienoic (EDA)	C20:2ω6	0.3 ± 0.1	0.4 ± 0.1	<0.001
γ‐Linolenic (GLA)	C18:3ω6	0.2 ± 0.1	0.2 ± 0.06	0.85
Dihomo‐g‐linolenic (DGLA)	C20:3ω6	0.5 ± 0.2	0.5 ± 0.1	<0.001
Arachidonic (AA)	C20:4ω6	0.4 ± 0.1	0.5 ± 0.1	<0.001
Docosatetraenoic (DTA)	C22:4ω6	0.1 ± 0.03	0.1 ± 0.03	<0.001
Docosapentaenoic‐n6 (DPA6)	C22:5ω6	0.09 ± 0.03	0.07 ± 0.02	<0.001
**∑** Cis‐ω6		13.3 ± 6.0	16.3 ± 3.5	<0.001
α‐Linolenic (ALA)	C18:3ω3	0.6 ± 0.5	1.7 ± 0.8	<0.001
Eicosapentaenoic (EPA)	C20:5ω3	0.07 ± 0.08	0.1 ± 0.08	<0.001
Docosapentaenoic‐n3 (DPA)	C22:5ω3	0.1 ± 0.07	0.2 ± 0.08	<0.001
Docosahexaenoic (DHA)	C22:6ω3	0.3 ± 0.1	0.4 ± 0.2	<0.001
∑ Cis‐ω3		1.1 ± 0.6	2.5 ± 1.0	<0.001
∑ Cis‐PUFA		14.4 ± 6.4	18.8 ± 3.9	<0.001
SFA/Cis‐PUFA		4.0 ± 1.8	2.5 ± 0.8	<0.001
Cis‐ω6/ ω3		13.4 ± 3.8	7.4 ± 2.5	<0.001
Palmitelaidic (PLA)	C16:1ω7t	0.05 ± 0.03	0.08 ± 0.04	<0.001
Elaidic (ELA)	C18:1 t	0.3 ± 0.2	0.4 ± 0.3	<0.001
Linoelaidic (LLA)	C18:2ω6t	0.3 ± 0.2	0.3 ± 0.1	0.66
∑ TFA		0.7 ± 0.3	0.8 ± 0.3	<0.001
Shannon entropy, *H*		1.9 ± 0.1	2.0 ± 0.1	<0.001

a Values are mean ± SD.

b %wt/wt of all FAs.

SFA, saturated fatty acids; Cis‐MUFA, Cis monounsaturated fatty acids, Cis‐PUFA, Cis polyunsaturated fatty acids; tFA, trans fatty acid.

Equality of mean between sites was tested by Welch's *t* test. A *p* value of .004 was considered significant with correction for multiple testing.

Given the nearly 33% estimated preterm rate in Bangladesh, albeit late prematurity, we tested whether this was associated with FA composition, and should be included as an explanatory term in multiple regression models. The univariate *t* tests of all 26 individual FAs and 12 major FA fractions for association with preterm gestational age at birth (<37 weeks) found 2 FAs that were significant with *p* value below or equal to .05 (stearic [STE], *p* value = .05; docosapentaenoic‐n6 [DPA6], *p* value = .03) but after correction for approximately 17 independent tests using the method described (Statistical Analyses), none were considered significant. We also tested for correlation of estimated gestational age at birth as a continuous variable with FA fraction. Similarly, STE and DPA6 were significant (STE, *p* value = .02; DPA6, *p* value = .04) but again were not significant after correction. Therefore, gestational age was not used as an adjustment in the multiple regression models.

The LASSO variable selection procedure was applied to penalized linear regression models of log (SFA/PUFA) and log (ω6‐PUFA/ω3‐PUFA) ratios, for Bangladesh and India separately (supplemental Figures 3–5). For log (SFA/PUFA) in Bangladesh, LASSO selected prosperity index 1 (*p* value less than .0001). In India, the first selected variable, mother's education level was not significant (*p* value = .14). For the log (ω6‐PUFA/ω3‐PUFA) outcome in Bangladesh, only the first selected variable, birth order of the infant, was significant (*p* value = .026) and in India, the first selected variable, total pregnancies, was also significant (*p* value = .019).

Having identified these variables from the LASSO selection, we carried them forward into joint multiple linear regression models together with the a priori selected maternal and SES variables. For log (SFA/PUFA), we found a significant negative association between prosperity index 1 in Bangladesh (Table 3) after adjusting for all prespecified variables, suggesting that increased family prosperity is associated with a relative decrease in SFA/PUFA ratio in breast milk. No association between prosperity index 1 and log ratio SFA/PUFA was observed at the Indian site. These relationships are shown in Figure 1. The mean value of prosperity index 1 was slightly but significantly higher in India (supplemental Table 6). Improved level of maternal education was negatively associated with log (SFA/PUFA) in Bangladesh (suggestive, *p* value = .04) and India (significant, *p* value = .004) after adjusting for other variables. Maternal height was also associated with this outcome in both sites, suggestive in Bangladesh (*p* value = .018) and significant in India (*p* value = .006), although the directions of effect were opposing. Each 1 cm of maternal height reduced the SFA/PUFA ratio by 1% in Bangladesh and increased by 1% in India. There was no independent association of maternal BMI with log SFA/PUFA or log ω6‐PUFA/ω3‐PUFA in either site.

**Table 3:** Association of maternal characteristics and least absolute shrinkage and selection operator (LASSO) selected predictors with log (saturated fatty acid /polyunsaturated fatty acid) in Bangladesh and India

	Effect	Multiplicative effect (95% CI)	*p* value	LASSO Penalized *p* value^a^
Bangladesh (*n* = 659)^b^				
Maternal age, year	0.005	1.00 (1.00, 1.02)	.30	‐
Maternal height, cm	−0.007	0.99 (0.99, 1.00)	.018	‐
Maternal BMI, kg/m^2^	−0.008	0.99 (0.98, 1.00)	.12	‐
Maternal education	−0.050	0.95 (0.91, 1.00)	.043	‐
Days of lactation	0.02	1.00 (1.00, 1.01)	.36	‐
Prosperity index1^c^	−0.058	0.94 (0.92, 0.96)	<.0001	<.0001
Birth order	−0.045	1.05 (0.99, 1.11)	.12	‐
India (*n* = 372)^b^				
Maternal age, year	−0.005	0.99 (0.98, 1.00)	.29	‐
Maternal height, cm	0.009	1.01 (1.00, 1.02)	.006	‐
Maternal BMI, kg/m^2^	0.007	1.01 (1.00, 1.02)	.11	‐
Maternal education	−0.074	0.93 (0.88, 0.98)	.004	‐
Prosperity index1	−0.004	1.00 (0.98, 1.01)	.63	‐
Total pregnancies	0.026	1.03 (0.98, 1.07)	.21	‐

a LASSO *p* values only shown for LASSO selected variables for this outcome.

b *n* values are the number of families with complete data.

c Prosperity index 1 was selected in the LASSO procedure for this outcome in Bangladesh, but was included in all models at both sites.

The tabulated variables were tested for association using Wald tests having jointly fitted all variables in an additive multiple linear regression model with intercept in each site.

Effect is per unit change in the predictor for the log ratio outcome.

Multiplicative effect is the effect converted to a multiplier of the nonlog ratio outcome.

For log (ω6‐PUFA/ω3‐PUFA), we found a negative association with birth order in Bangladesh (suggestive, *p* value = .018) and a similar negative association of total pregnancies with log (ω6‐PUFA/ω3‐PUFA) in India (*p* value = .010; Table 4). Because total pregnancies are correlated with birth order, and the same association was seen in the two closely related measures (gravidity and parity) in two independent cohorts in two different countries, this was considered to be evidence for a significant association. As seen in the univariate analyses of supplemental Table 5, days of lactation was significantly positively associated with log (ω6‐PUFA/ω3‐PUFA) in Bangladesh, as was prosperity index 1. The differences in complete data sample sizes in Bangladesh (*n* = 659) and India (*n* = 372) are unlikely to be the cause of any differences in conclusions for either outcome. With the effect sizes held constant, the increase in t‐statistic would be 1.33 times for India with a sample size of 659 equal to Bangladesh, and would not result in any differences in inference of statistically significant associations. We also tested for curvature in the association with prosperity index 1 in both outcomes in Bangladesh, but a single linear term was sufficient to explain the variation, excluding possible threshold effects within a site.

**Table 4:** Association of maternal characteristics and least absolute shrinkage and selection operator (LASSO) selected predictors with log (ω6‐polyunsaturated fatty acid/ω3‐polyunsaturated fatty acid) in Bangladesh and India

	Effect	Multiplicative effect (95% CI)	*p* value	LASSO Penalized *p* value^a^
Bangladesh *(n* = 659*)* b				
Maternal age, year	−0.001	1.00 (0.99, 1.01)	.69	‐
Maternal height, cm	−0.002	1.00 (0.99, 1.00)	.39	‐
Maternal BMI, kg/m^2^	−0.006	0.99 (0.99, 1.00)	.094	‐
Maternal education	−0.004	1.00 (0.97, 1.03)	.82	‐
Days of lactation	0.005	1.005 (1.00, 1.01)	.008	‐
Prosperity index1	0.019	1.02 (1.01, 1.03)	.003	‐
Birth order	−0.044	0.96 (0.92, 0.99)	.018	.026
India (*n* = 372)^b^				
Maternal age, year	0.0009	1.00 (0.99, 1.01)	.87	‐
Maternal height, cm	−0.004	1.00 (0.99, 1.00)	.27	‐
Maternal BMI, kg/m^2^	0.0004	1.00 (0.99, 1.01)	.95	‐
Maternal education	0.022	1.02 (0.97, 1.08)	.45	‐
Prosperity index1	0.011	1.01 (0.99, 1.03)	.28	‐
Total pregnancies	−0.063	0.94 (0.89, 0.98)	.010	.019

a LASSO *p* values only shown for LASSO selected variables for this outcome.

b *n* values are the number of families with complete data.

The tabulated variables were tested for association using Wald tests having jointly fitted all variables in an additive multiple linear regression model with intercept in each site.

Effect is per unit change in the predictor for the log ratio outcome.

Multiplicative effect is the effect converted to a multiplier of the nonlog ratio outcome.

## 4 DISCUSSION

We found that Bangladesh milk was higher in mean total percentage SFA (48%) than samples from India (44%), lower total PUFA, and disproportionately lower in ω3‐PUFA, such that the mean of ω6‐PUFA/ω3‐PUFA in Bangladesh was almost double that of India and close to the ratio observed in western countries (Simpoulos, 2002). Our analysis of factors associated with the SFA/PUFA and ω6‐PUFA/ω3‐PUFA found some common factors at the two sites; higher level of maternal education was associated with increased ratio of PUFA to SFA in breast milk sample; and increased birth order and total prior pregnancies associated with decreased ratio of ω6‐PUFA/ω3‐PUFA, or in other words, increased ratio of ω3‐PUFA relative to ω6‐PUFA. Of the site‐specific factors, in Bangladesh, household prosperity was independently associated with increased ratio of PUFA relative to SFA and increased ratio of ω6‐PUFA relative to ω3‐PUFA. In India, maternal height was associated with increased ratio of SFA relative to PUFA. Maternal BMI showed no independent association with any outcome in either site suggesting that adiposity is not independently associated with breast milk composition after controlling for other factors. The differences in the model results between the sites were not due to the difference in power from differing samples sizes, and after careful testing of all possible interactions of variables with sampling time during lactation in Bangladesh, we were able to reject more complex models and retain only a simple mean adjustment term for sampling. This means that we did not detect a change in the magnitude of effect of any of the associated maternal or SES variables at differing lactation stage.

The two cohorts in this study were recruited from low‐income neighborhoods in geographically close (150 miles) cities, that share West Bengali culture and ancestry, but in separate, bordering countries. Marine and freshwater fish constitute a comparatively larger proportion of protein intake in the Bengali diet, and both cities are riverine and near the coast. Of the common factors, we found that increased level of maternal education was associated with increased relative proportion of PUFA to SFA in breast milk. However, it did not influence the ω6‐PUFA/ω3‐PUFA distribution. We also found that birth order or total pregnancies was negatively associated with ω6‐PUFA/ω3‐PUFA ratio with a reduction of 0.95 per pregnancy or birth in both sites and was independent of maternal age or anthropometry. A study of rural Gambian breast milk samples showed a significantly higher percentage of ω6‐PUFA and a nonsignificant increase in percentage ω3‐PUFA from mothers with parity 10+ compared to primiparous (Prentice et al., 1989). The authors observed significantly lower endogenously produced FAs (C10:0, C12:0, and C14:0), which was compensated for by an increase in ω6‐PUFA. In well‐nourished Sudanese mothers, linoleic acid, PUFA and SFA increased with parity while 20:2 ω6 decreased (Laryea et al., 1995). However, these studies did not analyze the association of parity with the ω6‐PUFA/ω3‐PUFA ratio.

Despite the similarities, we found significant differences in mature breast milk composition, reiterating the importance of local or country‐specific factors. All of the ω3‐PUFA percentages were significantly higher among the Indian mothers than in Bangladesh, most likely resulting from a higher dietary fish intake (Parasuraman, Kishor & Vaidehi, 2008; Roy, Dhar, & Ghosh, 2012). The lower levels of PUFA in Bangladesh is accompanied by higher levels of SFA compared to India (48% compared to 43.7%) suggesting, perhaps, higher intake of carbohydrates. Bangladesh is also a high fish‐consuming nation, but the urban cohort we studied may have had more limited dietary choice because of economic or urban slum constraints. An earlier study among mothers of older infants in northern rural Bangladesh and another study among Iraqi mothers of different socioeconomic status noted a low intake of foods rich in PUFA among low‐income families (Al‐Tamer & Mahmood, 2006; Yakes et al., 2011), and this could explain the observed decreasing trend in SFA/PUFA with prosperity we also found.

Household prosperity was significantly associated with breast milk composition in Bangladesh but not in India, and while the mean prosperity in India was higher than Bangladesh, the range and distribution of prosperity indices was not very dissimilar between the sites, and there was considerable overlap of the lowest quartiles of prosperity in both sites. Because the prosperity index we developed was identically scaled in both sites and directly comparable, one explanation is that the cost of living in the urban site in Dhaka is higher than Kolkata such that foodstuffs with comparable PUFA content are more expensive. Dhaka has regularly ranked above Kolkata in global city cost of living rankings and in 2012, Dhaka ranked 184 versus Kolkata 208 (of 214 total cities; Mercer LLC, 2012).

Maternal height was found to be positively associated with increased SFA/PUFA ratio in India after adjustment for maternal age, education, BMI, birth order, and household prosperity, but the association in Bangladesh was in the opposite direction (suggestive, *p* value .018). The Bangladesh mothers were taller but with lower mean BMI, and greater percentage underweight than those in India (19%, Table 1). Possible explanations for this association include genetic pleiotropy of variants associated with FA metabolism also associated with height (Fumagalli et al., 2015), or cumulative dietary and/or health exposures over the first decades of life that affect linear growth and maternal metabolic reserves through epigenetic or other mechanisms. If the association with height is true in both sites, there may be two different mechanistic factors at work that lead to the bidirectional effect. Changes in the composition of dietary protein consumed has been shown to be associated with mean height by nationality (Grasgruber, Cacek, Kalina, & Sebera, 2014), and maternal nutritional status can affect the total lipid content of breast milk (Prentice & Prentice, 1995) or specific FA content (Antonakou et al., 2013). There is also evidence that poorly nourished mothers selectively retain essential FAs and their derivatives in their breast milk lipid fraction (Knox et al., 2000).

We compared the PROVIDE study breast milk FA results against those from a comparably large cohort study (*n* = 462) from a high‐income western country (Szabó et al., 2010). After dropping FAs that are not in our data and renormalizing to 100%, we derived approximate statistics for direct comparisons (supplemental Methods and supplemental Table 7). Interestingly, with exception of percentage AA and DTA, we observed strong evidence that percentages of all ω6‐ and ω3‐PUFAs were lower in breast milk from German mothers than in India and those in Bangladesh appeared closer to German mothers. Although percentage AA was not statistically different between Germany and either Bangladesh or India, percentage DHA was significantly higher in India and suggestively higher in Bangladesh. A recent meta‐analysis of 65 worldwide studies (Brenna et al., 2007) found that the percentage DHA in breast milk is more variable than percentage AA and comparing the values in our cohorts, we found that percentage AA was within the range seen in that study (mean = 0.47 ± 0.13%, range = 0.24 – 1.0%). Furthermore, Bangladesh mean percentage DHA was within the percentage DHA range (mean = 0.32 ± 0.22%, range = 0.06–1.4%), but India mean percentage DHA was significantly higher than the mean of the 65 studies (*p* value less than .001). These observations suggest that the breast milk composition in our two sites is at least generally comparable or even enriched for beneficial ω6‐ and ω3‐PUFAs compared to western levels, and that there are no obvious PUFA deficiencies, although optimal infant growth depends on an exquisite longitudinal balance of these micronutrients.

This is one of the largest single studies of fractional FA composition in breast milk published to date and one of the few ever published in populations drawn from the Indian subcontinent. This study is also the largest to date to use the novel and very convenient dried milk spot protocol for collection, storage, and shipment of large number of milk samples to a remote (overseas) laboratory for analysis. However, despite the large sample size, there are limitations to our study. Our analyses were based on an observational cohort and hence we cannot infer direct causation in our associated predictors. Although we have attempted to address possible biases in our analyses, there may be residual unobserved confounding. Our study was based on a single breast milk sample, which only partially captures the longitudinal changes of composition affecting infant growth. The Bangladesh samples were drawn over a range of lactation stages and infant ages up to 6_weeks postpartum, and although we were unable to detect a change in the effect sizes of the associated variables, it is possible that the changes in effects might not have been detectable because of the sample size. We did not collect information on dietary intake in the mothers. Although this does not invalidate the associations we found, maternal diet is likely to be a major factor in determining breast milk composition, and lack of this data limits our ability to infer mechanistic hypotheses for the associated variables. Lastly, samples were drawn from a specific geographical region of the countries and do not represent the low‐income populations of the countries as a whole.

In summary, our analysis of breast milk samples from two sites, Dhaka, Bangladesh and Kolkata, India showed that the socioeconomic factors of maternal education and household prosperity are associated with breast milk composition, although the latter was only a factor in Dhaka that has a higher cost of living. Associated maternal biological factors were height and infant birth order, but not adiposity. Further epidemiological and nutritional study is needed to elucidate the underlying mechanisms of these effects.

## SOURCE OF FUNDING

The Bill and Melinda Gates Foundation funded this work. The funding agency reviewed the design of the overall PROVIDE program and the inception of the cohorts. However, they had no role in the conduct of this study; data analysis; interpretation of the data; preparation of the manuscript; or in the decision of where or when to publish.

## CONFLICTS OF INTEREST

The authors declare that they have no conflicts of interest.

## CONTRIBUTIONS

The authors' responsibilities were as follows: WAP, BDK, RH, SK, and TFW designed the project; MA**,** ERC, SK, AD, DKP, SC, and SS clinical conduct of study; MPC, RKN, and WSH sample processing and lipid analysis; UN, BM, and DRK data management; DZ, JCM, and UN analyzed the data; UN and JCM wrote the paper; all authors performed the research and edited the paper.

## Supporting information

Supplemental Table 1. Inclusion and exclusion criteria in the PROVIDE study
Supplemental Table 2. Coefficients (loadings) of variables included in the principal component analysis measuring asset ownership and sources of wealth or prosperity in the household, for the first three principal components (prosperity index 1–3)
Supplemental Table 3. Correlation coefficients between prosperity indices and household expenditure by study site
Supplemental Table 4. The set of variables available for selection through the least absolute shrinkage and selection operator procedure in Bangladesh and India
