# Supplementary material for: Influence of maternal and socioeconomic factors on breast milk fatty acid composition in urban, low‐income families
Source: Matern Child Nutr. 2017 Feb 15;13(4):e12423. doi: 10.1111/mcn.12423 (PMC5638057; doi:10.1111/mcn.12423)
Supplement: Supplementary file 1 — Supplemental Table 1. Inclusion and exclusion criteria in the PROVIDE study Supplemental Table 2. Coefficients (loadings) of variables included in the principal component analysis measuring asset ownership and sources of wealth or prosperity in the household, for the first three principal components (prosperity index 1–3) Supplemental Table 3. Correlation coefficients between prosperity indices and household expenditure by study site Supplemental Table 4. The set of variables available for selection through the least absolute shrinkage and selection operator procedure in Bangladesh and India [file MCN-13-e12423-s001.docx]

**Online Supplemental Material**

**Influence of maternal and socioeconomic factors on breast milk fatty acid composition in urban, low-income families**

**by Nayak U. et al.**

**Supplemental Methods**

**1. LASSO Variable Selection and Multiple Regression Models**

The LASSO variable selection procedure was applied to penalized linear regression models of two outcomes:

1. log(SFA/PUFA)
2. log(ω6-PUFA/ω3-PUFA)

and was run separately for Bangladesh and India data. Since the LASSO algorithm selects a single variable from a clique of highly correlated variables, a single candidate was admitted into the test set as a proxy for the group of highly correlated variables. Both Bangladesh and India candidate variable sets included the first 3 prosperity indices. Variables were selected from LASSO using Mallow’s Cp statistic supported with covariance test of significance.

Having been selected in the LASSO procedure, the outcomes were tested in multiple linear regression models, for example:

$$\log\left( \frac{SFA}{PUFA} \right)=X\beta+e$$

for the log(SFA/PUFA) outcome where $X$ is a matrix of covariates and $\beta$ is the vector of coefficients.

**2. Correction for Multiple Testing of 26 Fatty Acids in Comparisons of Stage of Lactation (Bangladesh) and Bangladesh versus India Composition**

We corrected for multiple testing by estimating the effective number of independent tests conducted based on the correlation in the outcome fatty acid measures. For example, although we tested differences in composition between 26 fatty acids in the comparison of the Bangladesh and India samples, Figure 2 shows that at least some of the 26 x 25/2 fatty acid pairs are strongly correlated as measured by |r|, and others more weakly correlated but still not truly independent. We applied a method based on eigenvalue decomposition of the correlation matrix developed for genome-wide association studies (Supplemental Ref 1). The effective number of independent tests (*M_eff_*) is given by:

$$M_{eff}=26-\sum_{k=1}^{26} I\left\{ \lambda_{k}>1 \right\}\left( \lambda_{k}-1 \right) (1)$$

where I{ } is the indicator function and λ_k_ is the kth eigenvalue of the combined Bangladesh and India profiles (N=683+372=1055) correlation matrix.

**3. Inclusion of Bangladesh Samples in Comparisons with India and the Explanatory Multiple Linear Regression Models.**

As described in the main text and shown in Table 1, the Bangladesh breast milk samples were collected over a range of 2-43 days lactation, while the India samples were all mature milk collected between 42-49 days of lactation. In Table 2 in the main text we restricted the Bangladesh samples to 99 mature (/683 total) and compared with all 372 India samples.

The multiple regression explanatory models (Tables 3 and 4) tested the explanatory factors against two log ratio outcomes of major summary fractions (log (SFA/PUFA) and log (PUFA-6/PUFA-3). In these models were included all of the Bangladesh samples (N=683). The multiple regression models for Bangladesh included the sample days of lactation as an explanatory variable, and this also had the effect of adjusting the mean log outcome for variation in the sample time point. One major concern was that because the composition of the breast milk changes from colostrum to mature, the explanatory variables effects could change also. In fact as we showed in Supplementary Table 5 below, the log(SFA/PUFA) does not statistically significantly change between any of the three phases while the log(PUFA-6/PUFA-3) significantly increases. We were concerned that the effects of the included model variables (maternal age, height, bmi, education, prosperity index, total pregnancies or birth order) might change at different stages of lactation, and therefore we exhaustively tested for changes in the effects by adding every interaction term of the variable x days of lactation into the models to see if we could statistically detect a change in the effect of the variable over breast milk sample time point. None of these interaction terms for any variable reached the nominal 0.05 significance level in either the log(SFA/PUFA) or log(PUFA-6/PUFA-3) analyses, so that we rejected any model that explicitly corrected for changes in the effect by sample time point and only retained the sample time point as an adjustment to the mean.

**4. Comparison of the Fatty Acid Composition in PROVIDE Bangladesh and India Populations with the Germany Study of Szabó et al.**

The PROVIDE Bangladesh and India study results were also compared against previously published results from a reasonable sample size high-income country study (German cohort study, Szabó et al., 2010). Since the PROVIDE and German FA panels differed in composition, a direct comparison of compositions is not strictly appropriate but approximations were used to derive useful comparison results. Any FA that the German study did not assay was dropped from this FA panel and each maternal profile renormalized to 100%. Comparison also required the simplifying assumption that the 4 remaining FAs assayed in (Szabó et al., 2010) but not this study would not have altered the conclusions had this study included them in the panel. Since the Szabó study reported median and IQR values, the mean and standard deviation of their FAs was estimated by fitting the moments of the distributions. Tests of differences of mean FA fractions between studies used an approximate t-test interpreted conservatively; a t-statistic of 5-10 was treated as possible evidence, and >10 as strong evidence of difference in means between Germany and Bangladesh or India. For comparison a t-statistic of +/-3.3 corresponds to a 2-sided t-test p-value=0.001.

*Approximate Estimation of the Mean and Standard Deviation for the Szabó (Germany) Study*

Since the reported distribution parameter estimates from the Szabó study (Median, Inter-quartile Range IQR values) did not easily lend themselves to construction of a statistic to test either differences in means or medians between the PROVIDE study sites and the German samples, we attempted to fit their values to an assumed distribution to estimate the approximate and mean and stddev. We tested how well we could fit a beta distribution for each separate FA using estimates of the Median + IQR using our combined Bangladesh and India samples. Comparing the fits in our data with the actual estimated and mean and stddev, we found that the means were generally well fit but the stddev were substantially discrepant in multiple FAs. Hence we resorted to a simpler method of fitting. We fitted intercept-less regression models of the following form:

$Mean =cMedian$ (1)

$Stddev=kIQR$ (2)

using the combined the 26 x 2 = 52 FA parameter estimates from the separate Bangladesh and India data distributions and then assumed that the constants *c, k* are applicable to the FAs in the Szabó study also. We found that c = 1.004 +/- 0.006 indicating that the median is an unbiased estimator for the mean of the FA distributions as expected. The value k = 0.793 +/- 0.013. For comparison, if the 52 FA distributions were exactly normal, k = 1 /1.349 ~ 0.741.

To test for differences between the Szabó study FA distributions and those in Bangladesh or India, we constructed the following approximate t-statistic for each Fatty Acid:

$$\tilde{t}=\frac{\left( {Med}_{1}-{Med}_{2} \right)}{0.793\left[ \frac{{IQR}_{1}^{2}}{N_{1}}+\frac{{IQR}_{2}^{2}}{N_{2}} \right]^{1/2}} \left( 3 \right)$$

where subscript 1 = Szabó study and 2 = Bangladesh or India.

**Supplemental References**

1. Li MX, Yeung JM, Cherny SS, Sham PC. Evaluating the effective numbers of independent tests and significant p-value thresholds in commercial genotyping arrays and public imputation reference datasets. Hum Genet 2012;131(5):747-56. doi: 10.1007/s00439-011-1118-2.

**Supplemental Tables**

**Supplemental Table 1. Inclusion and exclusion criteria in the PROVIDE study**

| **Inclusion criteria** | |  |
| --- | --- | --- |
|  | **Bangladesh** | **India** |
| 1 | Mother willing to sign informed consent form | Mother willing to sign informed consent form |
| 2 | Healthy infant aged 0–7 days old | Healthy infant aged 0–49 days old |
| 3 | No obvious congenital abnormalities or birth defects | No obvious congenital abnormalities or birth defects |
| 4 | No abnormal (frequency and consistency) stools since birth |  |
| 5 | Stable household with no plans to leave the area for the next 1 year |  |
| **Exclusion criteria** | |  |
| 1 | Parents are not willing to have child vaccinated at the ICDDR,B field clinic |  |
| 2 | Parents are not willing to have child’s blood drawn | Parents are not willing to have child’s blood drawn |
| 3 | Parents are planning to enroll child into another interventional clinical study during the period of this trial that could affect the outcomes of this study | Parents are planning to enroll child into another clinical study during the time period of this trial |
| 4 | Mother not willing to have blood drawn and breast milk extracted | Mother not willing to have blood drawn and breast milk extracted |
| 5 | Parents not willing to have field research assistant in home two times per week | Parents not willing to have field research assistant in home |
| 6 | History of seizures or other apparent neurologic disorders | History of seizures or other apparent neurologic disorders |
| 7 | Infant received any vaccines before start of study, except BCG |  |
| 8 | Infant has any sibling currently or previously enrolled in this study, including a twin |  |
| 9 |  | Infant does not have proof of BCG and OPV since birth by immunization card |
| 10 |  | History of acute illness and/or immunocompromised state of the child |
| 11 |  | Immunocompromised or chronically ill mother |

**Supplemental Table 2. Coefficients (loadings) of variables included in the principal component analysis measuring asset ownership, and sources of wealth or prosperity in the household, for the first three principal components (prosperity index 1-3).**

| **Variable** | **Prosperity Index1** | **Prosperity Index2** | **Prosperity Index3** |
| --- | --- | --- | --- |
| Maternal Occupation | -0.021 | 0.059 | -0.002 |
| Principal drinking water source | -0.021 | 0.124 | -0.009 |
| Open drain beside your house | -0.006 | -0.024 | 0.053 |
| Place for cooking for household | 0.066 | -0.390 | -0.164 |
| In your dwelling is there Furniture: Cot or Bed | 0.097 | 0.018 | -0.164 |
| In your dwelling is there: Electricity | 0.102 | 0.176 | -0.355 |
| In your dwelling is there: Radio | 0.111 | -0.158 | -0.047 |
| In your dwelling is there: Bicycle | 0.112 | -0.254 | -0.269 |
| Principal type of toilet facility | 0.124 | -0.006 | -0.162 |
| In your dwelling is there: Bench | 0.125 | -0.035 | 0.116 |
| In your dwelling is there: Sewing machine | 0.146 | -0.031 | 0.017 |
| In your dwelling is there: Fan | 0.149 | 0.143 | -0.434 |
| Own the house you live in? | 0.166 | -0.270 | 0.060 |
| In your dwelling is there: Motor cycle | 0.166 | -0.089 | -0.099 |
| Household domestic worker | 0.183 | -0.026 | 0.269 |
| Principal wall material in your house | 0.184 | -0.277 | 0.069 |
| Principal type of flooring in your dwelling | 0.186 | 0.196 | -0.226 |
| In your dwelling is there: Phone | 0.192 | -0.001 | -0.211 |
| Principal roofing material in your house | 0.192 | 0.099 | -0.147 |
| Type of cooking fuel | 0.207 | 0.435 | -0.089 |
| In your dwelling is there: Cooking Gas | 0.216 | 0.421 | -0.034 |
| In your dwelling is there Furniture: Clock | 0.238 | -0.135 | -0.240 |
| In your dwelling is there: table | 0.242 | -0.019 | 0.266 |
| Paternal Occupation | 0.246 | 0.071 | 0.160 |
| In your dwelling is there: Television | 0.257 | -0.024 | -0.234 |
| Toilet facility shared with other households? | 0.265 | -0.159 | -0.226 |
| In your dwelling is there Furniture: Almeria | 0.275 | -0.158 | -0.054 |
| Number of rooms in current household | 0.288 | -0.186 | 0.159 |
| In your dwelling is there Furniture: Chair | 0.291 | -0.048 | 0.097 |

The variables are ordered by coefficient value in prosperity index 1 from most negative to most positive.

**Supplemental Table** **3. Correlation coefficients between prosperity indices and household expenditure by study site.**

| **Prosperity Index** | **Bangladesh** | **India** |
| --- | --- | --- |
| Prosperity Index 1 | 0.617 | 0.456 |
| Prosperity Index 2 | -0.082 | 0.133 |
| Prosperity Index 3 | 0.200 | 0.199 |
| Prosperity Indices 4 – 29, range | -0.05 – 0.197 | -0.159 – 0.139 |

For Prosperity Indices 4 – 29 the total range of the correlation coefficients is shown in summary. The correlation of prosperity index 1 with household expenditure in Bangladesh and India is shown graphically in Supplemental Figure 1, together with estimated correlation coefficient.

**Supplemental Table 4. The set of variables available for selection through the LASSO procedure in Bangladesh and India.**

| **No.** | **Variable** | **Notes** |
| --- | --- | --- |
| 1 | Maternal age | Also in pre-selected variable set |
| 2 | Maternal postpartum weight | Maternal bmi was used in the pre-selected variable set |
| 3 | Maternal height | Also in pre-selected variable set |
| 4 | Maternal education | Also in pre-selected variable set |
| 5 | Days of lactation | Also in pre-selected variable set |
| 6 | Birth order of infant |  |
| 7 | Total pregnancies |  |
| 8 | Household prosperity index 1 |  |
| 9 | Household prosperity index 2 |  |
| 10 | Household prosperity index 3 |  |
| 11 | Infant sex |  |
| 12 | Month when the breast milk sample collected |  |
| 13 | No of members of household |  |
| 14 | No of siblings < 5 yrs |  |
| 15 | How long lived in current household (yrs) | Not available in Bangladesh |
| 16 | No of people usually sleeping in household |  |
| 17 | Family type |  |
| 18 | Father’s education |  |
| 19 | Household monthly expenditure | Taka: Bangladesh; Rupees: India |
| 20 | Read newspaper? |  |
| 21 | Listen/watch radio/TV? |  |
| 22 | Water treatment method used |  |
| 23 | Household food availability |  |
| 24 | Hand washing before feeding infant |  |
| 25 | Hand washing before eating |  |
| 26 | Hand washing after defecating | Not available in India |
| 27 | Hand washing before cleaning infant’s bottle |  |
| 28 | Hand washing after cleaning infant’s anus | Not available in India |
| 29 | Frequency of mother nail cutting |  |

In Bangladesh, 28 total variables were available for selection while in India, 27 were available.

| **Supplemental Table 5. Percentage composition of breast milk fatty acid at different stages of lactation in Bangladesh*^a^*** | | | | | | | | | | | | | |  |  |
| --- | --- | --- | --- | --- | --- | --- | --- | --- | --- | --- | --- | --- | --- | --- | --- |
| **Fatty Acids***^b^* |  | **All samples** | | **Colostrum^c^** | | **Transitional^c^** | | **Mature^c^** | | **p-value** | |  |  |  |  |
| N |  | **683** | | **93** | | **491** | | **99** | |  | |  |  |  |  |
| Capric (CAP) | C10:0 | 1.2 ± 0.5 | | 0.6 ± 0.4 | | 1.2 ± 0.5 | | 1.3 ± 0.4 | | <0.001 | | |  |  |  |
| Lauric (LAU) | C12:0 | 8.2 ± 3.1 | | 5.9 ± 2.4 | | 8.5 ± 3.1 | | 8.5 ± 2.9 | | <0.001 | | |  |  |  |
| Myristic (MYR) | C14:0 | 8.0 ± 3.1 | | 7.8 ± 2.8 | | 8.1 ± 3.2 | | 7.8 ± 3.3 | | 0.98 | | |  |  |  |
| Palmitic (PAL) | C16:0 | 26.6 ± 3.7 | | 28.5 ± 3.4 | | 26.4 ± 3.7 | | 26.4 ± 4.0 | | <0.001 | | |  |  |  |
| Stearic (STE) | C18:0 | 3.9 ± 0.9 | | 4.2 ± 1.0 | | 3.9 ± 0.8 | | 3.9 ± 0.8 | | 0.015 | | |  |  |  |
| Arachidic (ARA) | C20:0 | 0.1 ± 0.03 | | 0.2 ± 0.04 | | 0.1 ± 0.03 | | 0.1 ± 0.03 | | 0.002 | | |  |  |  |
| Behenic (BEH) | C22:0 | 0.07 ± 0.02 | | 0.08 ± 0.02 | | 0.07 ± 0.02 | | 0.07 ± 0.03 | | <0.001 | | |  |  |  |
| Lignoceric (LIG) | C24:0 | 0.08 ± 0.03 | | 0.1 ± 0.04 | | 0.07 ± 0.02 | | 0.06 ± 0.02 | | <0.001 | | |  |  |  |
| ∑ **SFA^d^** |  | **48.2 ± 6.7** | | **47.3 ± 5.6** | | **48.5 ± 6.9** | | **48 ± 6.8** | | 0.44 | | |  |  |  |
| Palmitoleic (PLE) | C16:1*ω*7 | 2.9 ± 1.1 | | 2.6 ± 0.9 | | 3.0 ± 1.1 | | 3.0 ± 1.1 | | 0.017 | | |  |  |  |
| Oleic (OLE) | C18:1*ω*9 | 33.2 ± 4.6 | | 33.8 ± 4.5 | | 33 ± 4.6 | | 33.4 ± 4.9 | | 0.55 | | |  |  |  |
| Eicosenoic (EIC9) | C20:1*ω*9 | 0.4 ± 0.2 | | 0.6 ± 0.2 | | 0.4 ± 0.1 | | 0.4 ± 0.2 | | <0.001 | | |  |  |  |
| Nervonic (NER) | C24:1*ω*9 | 0.2 ± 0.1 | | 0.3 ± 0.2 | | 0.1 ± 0.08 | | 0.09 ± 0.07 | | <0.001 | | |  |  |  |
| ∑ ***Cis*-MUFA^d^** |  | **36.7 ± 5.1** | | **37.3 ± 4.7** | | **36.5 ± 5.1** | | **36.8 ± 5.2** | | 0.53 | | |  |  |  |
| Linoleic (LA) | C18:2*ω*6 | 11.3 ± 5 | | 10.9 ± 4.1 | | 11.2 ± 5.1 | | 11.7 ± 5.7 | | 0.28 | | |  |  |  |
| Eicosadienoic (EDA) | C20:2*ω*6 | 0.4 ± 0.2 | | 0.6 ± 0.2 | | 0.4 ± 0.2 | | 0.3 ± 0.1 | | <0.001 | | |  |  |  |
| γ-Linolenic (GLA) | C18:3*ω*6 | 0.2 ± 0.1 | | 0.1 ± 0.09 | | 0.2 ± 0.1 | | 0.2 ± 0.1 | | <0.001 | | |  |  |  |
| Dihomo-γ-linolenic (DGLA) | C20:3*ω*6 | 0.6 ± 0.2 | | 0.7 ± 0.2 | | 0.5 ± 0.2 | | 0.5 ± 0.2 | | <0.001 | | |  |  |  |
| Arachidonic (AA) | C20:4*ω*6 | 0.5 ± 0.2 | | 0.6 ± 0.2 | | 0.5 ± 0.1 | | 0.4 ± 0.1 | | <0.001 | | |  |  |  |
| Docosatetraenoic (DTA) | C22:4*ω*6 | 0.2 ± 0.1 | | 0.3 ± 0.2 | | 0.2 ± 0.07 | | 0.1 ± 0.03 | | <0.001 | | |  |  |  |
| Docosapentaenoic-n6 (DPA6) | C22:5*ω*6 | 0.1 ± 0.05 | | 0.2 ± 0.07 | | 0.1 ± 0.04 | | 0.09 ± 0.03 | | <0.001 | | |  |  |  |
| ∑ ***Cis*-*ω*6** |  | **13.2 ± 5.3** | | **13.4 ± 4.4** | | **13.2 ± 5.3** | | **13.3 ± 6.0** | | 0.95 | | |  |  |  |
| α-Linolenic (ALA) | C18:3*ω*3 | 0.5 ± 0.4 | | 0.5 ± 0.3 | | 0.5 ± 0.4 | | 0.6 ± 0.5 | | 0.20 | | |  |  |  |
| Eicosapentaenoic (EPA) | C20:5*ω*3 | 0.06 ± 0.07 | | 0.04 ± 0.03 | | 0.06 ± 0.07 | | 0.07 ± 0.08 | | 0.001 | | |  |  |  |
| Docosapentaenoic-n3 (DPA) | C22:5*ω*3 | 0.1 ± 0.08 | | 0.2 ± 0.1 | | 0.1 ± 0.07 | | 0.1 ± 0.07 | | <0.001 | | |  |  |  |
| Docosahexaenoic (DHA) | C22:6*ω*3 | 0.4 ± 0.1 | | 0.5 ± 0.2 | | 0.4 ± 0.1 | | 0.3 ± 0.1 | | <0.001 | | |  |  |  |
| ∑ ***Cis*-*ω*3** |  | **1.1 ± 0.5** | | **1.2 ± 0.4** | | **1.1 ± 0.5** | | **1.1 ± 0.6** | | 0.14 | | |  |  |  |
| ∑ ***Cis*-PUFA^d^** |  | **14.4 ± 5.7** | | **14.6 ± 4.7** | | **14.3 ± 5.7** | | **14.4 ± 6.4** | | 0.85 | | |  |  |  |
| **SFA/Cis-PUFA** |  | **3.9 ± 1.7** | | **3.6 ± 1.3** | | **4.0 ± 1.7** | | **4.0 ± 1.9** | | 0.12 | | |  |  |  |
| ***Cis*-*ω*6/ *ω*3** |  | **12.3 ± 3.3** | | **11.7 ± 2.7** | | **12.3 ± 3.2** | | **13.3 ± 3.8** | | <0.001 | | |  |  |  |
| Palmitolaidic (PLA) | C16:1*ω*7t | 0.06 ± 0.03 | | 0.07 ± 0.04 | | 0.06 ± 0.03 | | 0.05 ± 0.03 | | <0.001 | | |  |  |  |
| Elaidic (ELA) | C18:1t | 0.4 ± 0.3 | | 0.4 ± 0.3 | | 0.4 ± 0.3 | | 0.3 ± 0.2 | | <0.001 | | |  |  |  |
| Linoelaidic (LLA) | C18:2*ω*6t | 0.3 ± 0.2 | | 0.3 ± 0.2 | | 0.3 ± 0.2 | | 0.3 ± 0.2 | | 0.66 | | |  |  |  |
| ∑ ***TFA^d^*** |  | **0.7 ± 0.4** | | **0.8 ± 0.4** | | **0.7 ± 0.4** | | **0.7 ± 0.3** | | 0.013 | | |  |  |  |
| Shannon Index, H |  | **1.9 ± 0.1** | | **1.9 ± 0.1** | | **1. 9 ± 0.1** | | **1.9± 0.1** | | 0.33 | | |  |  |  |
| ***^a^*** Values are mean ± SD rounded to 1 decimal place or 1 sig. fig. if < 0.1; | | | | | | | | | | | | | | |  |
| ***^b^*** %wt/wt of all FAs; |  | |  | |  | |  | |  | |  | | | | |
| ^c^ Colostrum < 5 days; Transitional 6 – 15 days; Mature > 15 days;  ^d^ SFA, Saturated fatty acids; *Cis*-MUFA, *Cis* monounsaturated fatty acids, *Cis*-PUFA, *Cis* polyunsaturated fatty acids; *t*FA, *trans* fatty acid | | | | | | | | | | | | | |  |  |
| Differences between lactation group means were tested using one-way ANOVA with Welch’s heteroskedasticity correction if Barlett’s test showed non-equal variances. A p-value of 0.004 is considered significant with correction for multiple testing. | | | | | | | | | | | | | |  |  |

**Supplemental Table 6. Comparison of the first three prosperity indices in Bangladesh and India**

|  |  |  |  |  |
| --- | --- | --- | --- | --- |
| **Prosperity Index** | **Bangladesh** | **India** | **p-value** | |
| Prosperity Index 1 | -0.18 | 0.32 | <0.0001 | |
| Prosperity Index 2 | 0.72 | -1.27 | <0.0001 | |
| Prosperity Index 3 | 0.29 | -0.52 | <0.0001 | |

The value of the mean prosperity index variable is shown for the cohort in each site, together with the p-value for a t-test of difference between the means

| **Supplemental Table 7. Comparison of the common breast milk fatty acids between the PROVIDE study sites and the Szabo (Germany) study*^a^*** | | | | | | | | |  |
| --- | --- | --- | --- | --- | --- | --- | --- | --- | --- |
| **Fatty Acid*^b^*** |  | **Germany** | **Bangladesh*^c^*** | **BD**  **t-stat**^d^ | | | **India** | **India**  **t-stat**^d^ | |
| n |  | 462 | 99 |  | | | 372 |  | |
| **SFA** |  |  |  |  | | |  |  | |
| Capric(CAP) | C10:0 | 2.1 (1.3) | 1.2 (0.5) | **14.2** | | | 0.9 (0.6) | **22.2** | |
| Lauric(LAU) | C12:0 | 6.1 (3.1) | 8.2 (3.4) | -6.8 | | | 6.4 (3.2) | -1.2 | |
| Myristic (MYR) | C14:0 | 7.0 (2.8) | 7.1 (3.6) | -0.2 | | | 7 (3.6) | 0.2 | |
| Palmitic(PAL) | C16:0 | 22.6 (3.8) | 26.9 (5.2) | -9.8 | | | 23.8 (3.9) | -5.8 | |
| Stearic(STE) | C18:0 | 7.8 (2.3) | 3.8 (0.7) | **39.4** | | | 4.2 (0.8) | **38.7** | |
| Arachidic(ARA) | C20:0 | 0.3 (0.1) | 0.1 (0.04) | **20.2** | | | 0.2 (0.07) | **11.2** | |
| Behenic(BEH) | C22:0 | 0.1 (0.08) | 0.06 (0.03) | **18.4** | | | 0.09 (0.04) | **11.8** | |
| Lignoceric(LIG) | C24:0 | 0.1 (0.1) | 0.06 (0.02) | **11.1** | | | 0.1 (0.02) | **12** | |
| **MUFA** |  |  |  |  | | |  |  | |
| Palmitoleic(PLE) | C16:1n7 | 2.7 (1.2) | 3.0 (1.4) | -2.6 | | | 2.3 (1.1) | 5.2 | |
| Oleic(OLE) | C18:1n9 | 30.9 (5.7) | 33.7 (7.3) | -4.5 | | | 32.6 (5) | -5.6 | |
| Eicosenoic(EIC9) | C20:1n9 | 0.3 (0.2) | 0.3 (0.09) | 2.0 | | | 1 (0.7) | **-22.3** | |
| Nervonic(NER) | C24:1n9 | 0.06 (0.09) | 0.1 (0.04) | -4.6 | | | 0.5 (0.3) | **-31.3** | |
| **ω6-PUFA** |  |  |  |  | | |  |  | |
| Linoleic (LA) | C18:2n6 | 10.1 (4.4) | 9.2 (6) | 1.7 | | | 14.3 (4.3) | **-17.6** | |
| γ-Linolenic (GLA) | C18:3n6 | 0.1 (0.1) | 0.2 (0.1) | -2.8 | | | 0.2 (0.07) | **-11.1** | |
| Eicosadienoic(EDA) | C20:2n6 | 0.2 (0.1) | 0.3 (0.1) | -4.5 | | | 0.4 (0.1) | **-27.2** | |
| Dihomo-γ-linolenic (DGLA) | C20:3n6 | 0.3 (0.2) | 0.5 (0.2) | **-10.4** | | | 0.5 (0.2) | **-13.5** | |
| Arachidonic (AA) | C20:4n6 | 0.5 (0.3) | 0.4 (0.1) | 2.5 | | | 0.5 (0.1) | -1.2 | |
| Docosatetraenoic (DTA) | C22:4n6 | 0.06 (0.1) | 0.1 (0.03) | **-11.4** | | | 0.1 (0.04) | -9.9 | |
| **ω3-PUFA** |  |  |  |  | | |  |  | |
| α-Linolenic (ALA) | C18:3n3 | 0.7 (0.4) | 0.4 (0.5) | 7.9 | | | 1.5 (1.0) | **-19** | |
| Eicosapentaenoic (EPA) | C20:5n3 | 0.04 (0.07) | 0.05 (0.05) | -2.1 | | | 0.1 (0.08) | **-16.2** | |
| Docosapentaenoic - n3 (DPA) | C22:5n3 | 0.07 (0.2) | 0.1 (0.08) | | -4.0 | 0.2 (0.1) | | **-14.8** | |
| Docosahexaenoic (DHA) | C22:6n3 | 0.2 (0.2) | 0.3 (0.1) | | -8.8 | 0.4 (0.2) | | **-17.2** | |
| **TFA** |  |  |  | |  |  | |  | |
| Palmitolaidic(PLA) | C16:1n7t | 0.3 (0.2) | 0.05 (0.04) | | **36.1** | 0.08 (0.05) | | **34.6** | |
| Linoelaidic(LLA) | C18:2n6t | 0.3 (0.2) | 0.3 (0.1) | | 0.0 | 0.3 (0.1) | | -2.7 | |
| ***^a^***Values are median (IQR) rounded to 1 decimal place or 1 sig. fig. if < 0.1; | | | | | | | | |  |
| ***^b^*** %wt/wt of all FAs; | | | | | | | | |  |
| *^c^* Bangladesh restricted to only mature breast milk samples > 15 days of lactation;  ^d^ BD t-stat, Bangladesh approximate t statistic; India t-stat, India approximate t statistic; values in bold in these two columns indicates a t-statistic > 10 and strong evidence of difference in means of that FA in Germany and the PROVIDE site; t-statistics that are +ve in sign indicate that German samples have a higher mean level of that FA than the PROVIDE site, and vice versa. | | | | | | | | |  |

**Supplemental Figures**

**693**

**7 – Consent withdrawn**

**372**

**683**

**372**

**10 – Sample not sufficient**

**Breast milk samples collected**

**Breast milk samples assayed**

**Bangladesh India**

**Enrolled**

**700**

**372**

**Supplemental Figure 1.** Study flowchart of breast milk sample collection and fatty acid analysis in Bangladesh and India cohorts.

**Supplemental Figure 2.** Correlation heat maps of major fatty acid components in Bangladesh and India breast milk samples. The correlations were calculated using all 683 breast milk samples in Bangladesh (all lactation stages) and all 372 in India (mature sampling only).

**Supplemental Figure 3.** The LASSO variable selection path for the log(SFA/PUFA) outcome for Bangladesh and India. The y-axis displays Mallow’s Cp index after each new variable is selected into the penalized regression model, while the x-axis (df) shows the number of variables selected (degrees of freedom). The first degree of freedom corresponds to the overall mean of the outcome, so that df=2 is the first independent variable selected, df=3 is the second and so on. The first distinct inflection point in the Bangladesh data is at df=2; in India it also occurs at df=2 but the change in Cp is relatively smaller. In Bangladesh, the first variable selected was the family prosperity index1 (LASSO covariance test p-value <0.0001) while the second variable, water source, was not significant (p-value=0.5). In India, the first variable, mother’s education level was not significant (p-value=0.14).

**Supplemental Figure 4.** The LASSO variable selection path for the log(ω6-PUFA/ω3-PUFA) outcome for Bangladesh. The axes and interpretation of the figure are as described in the Supplemental Figure 2 legend. The first variable selected in Bangladesh, birth order of the infant (df=2), was significant under the covariance test (p-value=0.026) but the second variable (df=3) was not significant (p-value=0.14).

**Supplemental Figure 5.** The LASSO variable selection path for the log(ω6-PUFA/ω3-PUFA) outcome for India. The axes and interpretation of the figure are as described in the Supplemental Figure 2 legend. Only the first selected variable, total pregnancies, was significant (p-value=0.019).
